# Delayed processing of global shape information in developmental prosopagnosia

**DOI:** 10.1371/journal.pone.0189253

**Published:** 2017-12-20

**Authors:** Christian Gerlach, Solja K. Klargaard, Anders Petersen, Randi Starrfelt

**Affiliations:** 1 Department of Psychology, University of Southern Denmark, Odense, Denmark; 2 Department of Psychology, University of Copenhagen, Copenhagen, Denmark; University of Akron, UNITED STATES

## Abstract

There is accumulating evidence suggesting that a central deficit in developmental prosopagnosia (DP), a disorder characterized by profound and lifelong difficulties with face recognition, concerns impaired holistic processing. Some of this evidence comes from studies using Navon’s paradigm where individuals with DP show a greater local or reduced global bias compared with controls. However, it has not been established what gives rise to this altered processing bias. Is it a reduced global precedence effect, changes in susceptibility to interference effects or both? By analyzing the performance of 10 individuals with DP in Navon’s paradigm we find evidence of a reduced global precedence effect: The DPs are slower than controls to process global but not local shape information. Importantly, and in contrast to previous studies, we demonstrate that the DPs perform normally in a comprehensive test of visual attention, showing normal: visual short-term memory capacity, speed of visual processing, efficiency of top-down selectivity, and allocation of attentional resources. Hence, we conclude that the reduced global precedence effect reflects a perceptual rather than an attentional deficit. We further show that this reduced global precedence effect correlates both with the DPs’ face recognition abilities, as well as their ability to recognize degraded (non-face) objects. We suggest that the DPs’ impaired performance in all three domains (Navon, face and object recognition) may be related to the same dysfunction; delayed derivation of global relative to local shape information.

## Introduction

Developmental prosopagnosia (DP) is a disorder characterized by profound and lifelong difficulties with face recognition in the absence of any sensory or intellectual deficits or known brain injury [1]. Whether the disorder is selective for faces, or whether it may also affect the visual processing of other categories of objects beside faces, is debated [2–4]. What does seem clear, and what is a precondition for the diagnosis, is that everyday recognition of faces is far more affected than recognition of other types of objects. Indeed, individuals with DP can report numerous examples of experiences in which they were unable to recognize individuals after exposure that would lead to recognition success in neurologically typical people [1]. In our experience, they do not report similar problems with other categories of objects.

What can possibly be the underlying cause of this disorder? Given that all faces share the same features (nose, eyes etc.), arranged in a similar manner (the nose is located under the eyes and over the mouth; so called first-order relations), face recognition may depend on the identification of the exact shape of these features, the metric relationship between these features, so called second-order relations [5, 6], and probably in particular on the binding of these two types of information. This suggests that efficient face recognition may necessitate some sort of holistic processing wherein all these aspects are integrated. There are several lines of evidence supporting the notion that DP may reflect a problem in holistic processing [7]. We will briefly describe two of them here, the Garner interference effect [8] and The Composite Face effect [9], before we turn to Navon’s paradigm which is the main focus of the present investigation.

The Garner interference effect is based on Garner’s speeded-classification paradigm [10]. This paradigm examines whether an observer can attend to one dimension of an object without experiencing interference from task irrelevant variation on another dimension. If they can, these dimensions are separable. If they cannot, there will be an interference effect, suggesting that the dimensions are integral (holistically processed). This paradigm has been used by Amishav and Kimchi [8] to examine whether individuals can process the features in faces without being affected by changes in the second-order relations of the same features and vice versa. As an example; does a change in the spacing between the eyes (second-order dimension) affect our judgment of potential changes in the features themselves (feature dimension)? Apparently it does, as task irrelevant variation gives rise to symmetrical interference effects, so that variation in features affects the judgment of variation in second-order relations and vice versa. In comparison, individuals with DP exhibit no Garner interference along these dimensions, suggesting a break-down of integral (holistic) processing [11].

In the Composite Face Task the observer is presented with two faces (either simultaneously or delayed), and asked to decide whether the two top-halves are identical or not (ignoring the bottom-halves of the faces). It turns out that people find this task difficult and often report that the top-halves are different when in fact only the bottom-halves are. This is not merely a Stroop like interference effect [12], reflecting that the stimuli are incongruent in some respects, because the increase in error rate/reaction time disappears, or is greatly reduced, when the two halves of the faces are spatially offset (misaligned). Hence, when the face-gestalt is disturbed by misalignment, there is a release from interference even when the incongruent information is still present. Like the Garner interference effect, the Composite Face effect also suggests that people cannot refrain from processing parts of faces even when these parts are task irrelevant. Regarding how individuals with DP perform on the Composite Face Task, the picture is mixed. While some studies have found that individuals with DP show no or a reduced interference effect [13], and that there may even be a positive correlation between the magnitude of the interference effect and performance on standard face recognition tasks [2], others have reported normal interference effects [14–17].

Despite some inconsistencies, the studies considered above seem to suggest that face recognition is based on holistic processing, and that this type of processing may be impaired in individuals with DP [18]. This agreement regarding impaired holistic processing in DP, however, may be more apparent than real as several different definitions of the construct ‘holistic’ can be found in the literature [19]. On one account, holistic processing is thought to reflect that faces cannot (easily) be decomposed into parts because faces are represented as undifferentiated wholes where the parts do not have a distinct representation (e.g., Tanaka and Farah [20]). A more liberal version of this proposition has been advanced by Rossion [21]. He argues that parts of faces are processed simultaneously, guided by stored representations which are inherently holistic (templates). However, the parts in these holistic representations do not need to have the same weight in the recognition process. As an example, people may be more affected by changes to the eye than the nose region even if both regions are processed simultaneously. Accordingly, in this liberal version, parts must to some extent be represented distinctly even if the representation acts as a whole. A different view has been expressed by Behrmann, Avidan [22]. They seem to equate ‘holistic’ with representations placing special weight on (second-order) configural relations among the features. In later writings, this emphasis on second-order relations has been downplayed somewhat by giving equal weight to features and second-order relations. Thus, these authors now reserve the term ‘holistic’ to the integration of features and their configuration [11].

Common for the interpretations of holistic processing considered thus far is their perceptual nature; they primarily concern the representational substrate of face recognition. However, the deficit underlying DP could in principle be strictly attentional in nature. Imagine for example that the visual processing capacity is somehow reduced in DP, so that visual elements can no longer be processed in parallel. In this case, attentional allocation would take on a more serial mode in which single features (or small groups of nearby features) have to be processed in turn. An individual with such a disorder would clearly be less susceptible to the Composite Face effect or Garner interference effects, and this regardless of whether face representations in visual long-term memory are stored as (holistic) templates or not. In line with this, Richler, Palmeri [19] have stated that: “At present, holistic processing as a failure of selective attention seems the strongest candidate” (p. 4). While we agree with these authors’ point that neither the Composite Face effect or the Garner interference effect in themselves point to a specific impairment (or version of ‘holistic’), we do find it to be a misnomer calling such effects the result of a failure of selective attention. Taken literally, it would imply that it is the individuals with DP who have normal ‘selective’ attention, whereas individuals with normal face recognition—who are susceptible to interference effects—have impaired selective attention.

The main point made by Richler, Palmeri [19] still highlights the need for examining visual attention proper in individuals with DP; just as it is important to examine their general visual object recognition abilities. Without information concerning how individuals with DP perform in these (related) domains, it will be difficult to come closer to an understanding of why face recognition fails in DP.

### The relationship between Navon performance and face recognition

As opposed to the studies considered above, the results to be presented here are based on Navon’s paradigm. For this reason we will briefly discuss how findings based on this paradigm may relate to face processing.

Navon’s paradigm [23] involves the presentation of compound stimuli; typically large letters (global level) composed of smaller letters (local level) in which the global and the local letters may be the same (consistent) or different (inconsistent). While different effects may be obtained with this paradigm depending on exposure duration, masking, letter spacing, stimulus clarity, eccentricity, or attentional demands (divided or selective) [24–26], three effects are usually found: (i) a *global precedence effect* with faster judgements of the identity of the global shape compared with the local elements, (ii) an *interference effect* with slower responses to inconsistent than consistent stimuli, and (iii) an *inter-level interference effect* with greater interference effects on local compared with global identity trials.

If failures in face recognition reflect not only impaired processing of features but also impaired processing of the configuration of these features, it is probable that individuals with DP may also experience difficulties in this paradigm. After all, the global precedence effect suggests that the overall configuration of the local elements is derived and recognized before the identity of the local elements is resolved. Hence, difficulties in deriving the relationship between features may be reflected in the Navon paradigm as a reduced global precedence effect or even a local precedence effect [2, 22].

While not all studies have found a correlation between face recognition ability and performance in Navon’s paradigm in DP [27], some have. Behrmann, Avidan [22] reported a marginally significant correlation (*r* = .84, *p* = .07) between face discrimination performance and a global/local index derived from the Navon paradigm [(Local-Global Inconsistent trials)–(Local-Global Consistent trials)] in a small group of individuals (*N* = 5): The greater the local bias, the poorer the performance in the face discrimination task. Using a larger sample of individuals (*N* = 13), Avidan, Tanzer [2] found a marginally significant correlation (*r* = .52, *p* = .06) between the same Navon index and performance in the Composite Face paradigm: The greater the local bias, the less effect of interference in the Composite Face task. Duchaine, Yovel [28] examined how a variety of indexes derived from the Navon paradigm correlated with performance on the Cambridge Face tests in a relatively large sample of individuals with DP (*N* = 14). While most of these indexes did not correlate with face recognition performance, one did: Their measure of Global-to-local interference [(Local Consistent–Inconsistent trials) / (Local Consistent+Inconsistent trials)] correlated (*r* = .58, *p* = .03 uncorrected for multiple comparisons) with performance on the Cambridge Face Memory Test (CFMT; Duchaine and Nakayama [29]).

It is evident from this short review, that studies relating performance in Navon’s paradigm to face recognition ability have been based on different indexes derived from the Navon paradigm.

Some of these indexes are complex in that they tap inter-level interference effects [2] while others are more simple in that they tap global/local precedence effects or interference effects confined to a particular level [28]. Considering that global/local precedence effects and inter-level interference effects reflect different mechanisms [30, 31], it is perhaps not surprising that different results have been obtained. Furthermore, the indexes applied also seem to differ considerably in reliability [30], which may have added further to the discrepancies.

In the present study we thus decided to use an index of global/local processing that was both simple and reliable, and that would allow us to examine whether face recognition in DP is systematically related with a reduced or even reversed global precedence effect. This index, which we term the ‘Global-Local Precedence index’, is based on the standardized mean difference (Cohen’s *d*) between reaction times (RT) to Local and Global Consistent trials. The index is simple because it measures differences in global and local processing unconfounded by interference effects. Based on the data presented in Gerlach and Krumborg [30] we computed the Spearman-Brown-corrected split-half reliability of this index to be .78.

We also tested the general visual attentional capacity of the DPs using a combination of psychophysical experiments and mathematical data modelling based on the Theory of Visual Attention (TVA) (Bundesen, 1990). This was done to examine whether potential abnormalities on Navon’s paradigm might reflect attentional deficits.

## The Navon experiment

### Method

#### Participants

Background information on the group of individuals with DP (*N* = 10) we test here has previously been reported by Gerlach, Klargaard [4]. All of these individuals performed significantly outside the normal range on the Cambridge Face Memory Test and the first part of the Faces and Emotion Questionnaire (29-items) [32] compared with a matched control sample. The mean age of the DPs was 37 (range: 16–57). Six of them are females. All participants provided written informed consent according to the Helsinki declaration. The Regional Committee for Health Research Ethics of Southern Denmark has assessed the project, and ruled that it did not need formal registration. For the specific participant who was 16 years at the time of the investigation, both a parent and the participant were provided with written information about the project, and the participant then signed the consent form with the knowledge of the parent.

The DPs’ performance in Navon’s paradigm is compared with 20 control participants, two for each individual with DP, matched for age and gender. We decided to exclude two of the original 20 controls because they exhibited unusually high error rates in Navon’s paradigm (14 and 23%). The excluded controls were replaced with two other matched controls (Mean age in the adjusted control group = 37, range: 16–56). This did not alter the results to be reported.

#### Design

The participants were presented with large letters, either ‘H’ or ‘S’, that could consist of either smaller ‘H’s or ‘S’s. The experiment comprised four experimental blocks. In two blocks, the participants were required to report the identity of the large (global) letter, whereas they were to report the identity of the small (local) letters in the other two blocks (a selective attention paradigm). The blocks were presented in an ABBA design beginning with global identity judgements.

#### Stimuli

The large letters were 4.1 cm wide and 5.5 cm high (3.91° × 5.25°) and the small letters were 0.5 cm wide and 0.7 cm high (0.47° × 0.67°). The fixation cross presented before stimulus onset was 1 cm wide and 1 cm high (0.95° × 0.95°). All stimuli were black presented on a white background on a computer screen.

#### Procedure

Participants performed a total of 48 trials in each block, 24 consistent (same identity of local and global elements) and 24 inconsistent (different identity of the local and global elements). The stimuli were shown at either the right or the left side of the fixation cross, with the centre of the global shape positioned 3.5 cm (3.34°) from the fixation cross. An equal number of stimuli within each block (*n* = 24: 12 consistent and 12 inconsistent) were presented to the right and the left. The order of position and consistency (consistent vs. inconsistent stimuli) was randomized. A trial began with a fixation cross presented in the middle of the screen for 1 s, which the participants were instructed to look at when present. This was followed by stimulus onset which was replaced after 180 ms. by a blank screen which remained until response. Responses were recorded via a serial response box placed in front of the participants’ right hand. Before each block, the participants performed 16 practice trials. Feedback was provided during the practice trials but not during the experimental blocks.

#### Statistical analyses

Prior to statistical analysis data were trimmed excluding any RT falling 2.5 SD’s beyond or below the individual means for either consistent or inconsistent trials at each presentation location (left and right) and at each level (global and local); i.e., trimming was performed across a total of eight individual means for each participant. The trimming resulted in an average of 3% trials removed for the DP group (range: 0.5–4.7%). This was rather similar to the control participants (average = 2.2%; range: 0–4.2%). Both figures are well within the recommended limits suggested by Ratcliff [33].

As described above, one of the objectives of the present study is to examine whether face recognition in DP is systematically related to the Global-Local Precedence index derived from Navon’s paradigm; an index which is based on the standardized mean RT to Local and Global Consistent trials. Considering the relatively small sample size of the DP group we also examined whether the DP group and control group differed reliably on this index using robust statistics by estimating the 95% CI of the mean difference between groups based on bias corrected bootstrap analysis (1000 samples).

### Results

#### Error rates

The DP group made 1.8% errors on average (range: 0–4.2%) whereas the control participants made an average of 2.6% (range: 0–6.8%). Comparing the error rates for the DP and the control groups for each of the four conditions (Global Consistent, Global Inconsistent, Local Consistent, and Local Inconsistent) did not reveal any significant differences (Mann-Whitney exact test, all *p*’s > .1) (See also S1 Dataset).

#### RT

The mean correct RTs were subjected to a mixed factorial ANOVA with the factors Group (DPs vs. controls), Level (global [large letter] vs. local [small letter] shape judgments), and Consistency (consistent vs. inconsistent trials). This analysis revealed a significant main effect of Consistency (*F*(1,28) = 40.2, *MS*_*e*_ = 80374, partial η^2^ = .59, *p* < .001), with faster responses on consistent trials, and a significant three-way interaction between Group, Level and Consistency (*F*(1,28) = 6.03, *MS*_*e*_ = 9275, partial η^2^ = .18, *p* < .05). The three-way interaction was further examined using separate within-subject ANOVA’s with Group as the separating variable.

**Fig 1:**
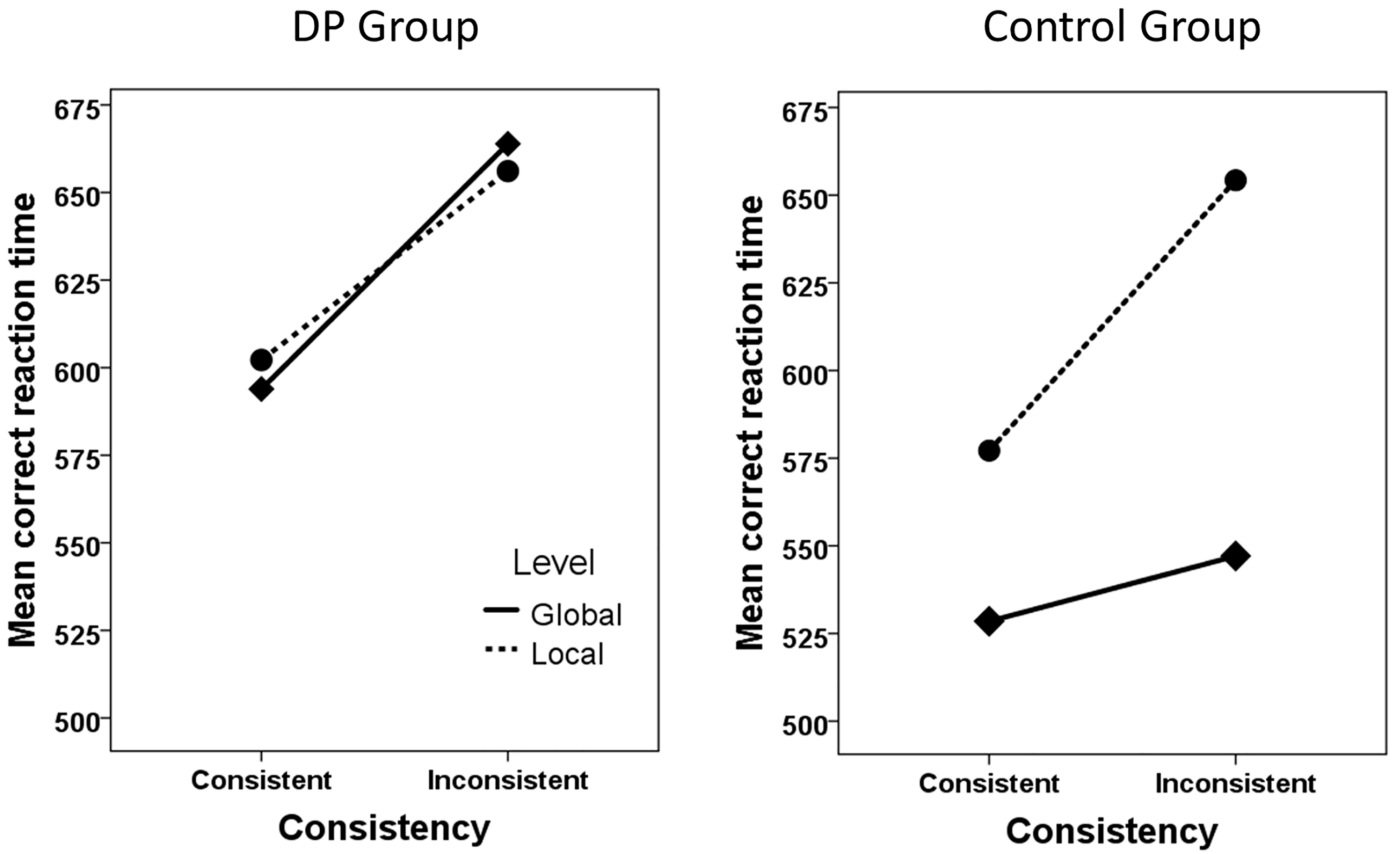
Illustration of the performance for the DP group and the control group in the four conditions of the Navon paradigm.

The ANOVA for the DP group revealed a significant main effect of Consistency (*F*(1,9) = 8.9, *MS*_*e*_ = 38378, partial η^2^ = .50, *p* < .05). No other effects were significant (all *p*’s > .6). In comparison, the ANOVA for the control group revealed a significant main effect of Level (*F*(1,19) = 44.9, *MS*_*e*_ = 121368, partial η^2^ = .70, *p* < .001), with faster identity judgments of global shapes, a significant main effect of Consistency (*F*(1,19) = 50.3, *MS*_*e*_ = 45792, partial η^2^ = .73, *p* < .001), and a significant interaction between Level and Consistency (*F*(1,19) = 14.6, *MS*_*e*_ = 17111, partial η^2^ = .44, *p* < .01). Simple main effects analysis (paired samples *t*-test) of this interaction revealed that all simple main effects were significant (all *p*’s < .01). See Table 1 and Fig 1 for details concerning mean correct RTs and SDs (see also S1 Dataset for additional information regarding the individual scores on Navon’s paradigm).

**Table 1:** Performance in Navon’s paradigm.

	Global Consistent	Global Inconsistent	Local Consistent	Local Inconsistent
**DPs**	594 (144)	664 (241)	602 (132)	656 (107)
**Controls**	529 (144)	547 (142)	577 (148)	654 (164)

Mean correct RT and SD (in brackets) for the group of individuals with developmental prosopagnosia (DP) and the control group in the four conditions of the Navon paradigm.

#### Effect sizes

The mean standardized difference for the DP group between RTs to Local and Global Consistent trials (the Global-Local Precedence index) was .08. In comparison it was .54 for the control group. This difference was reliable (*M*_*dif*_ = .46, 95% CI [.09 - .93], *p* < .05). As will be discussed below, one of the DPs (DP18) obtained an extreme score on the Global-Local Precedence index (-1.76). Hence, we redid the analysis removing her and her two controls from the analysis. This did not alter the results as the difference between the DP and control group was still reliably different (*M*_*dif*_ = .35, 95% CI [.11 - .62], *p* < .05). For additional information regarding the individual scores on the Global-Local Precedence index see S1 Dataset.

### Discussion

The results from the Navon paradigm seem clear: While both groups exhibit a *consistency effect*, with faster responses on consistent compared with inconsistent trials, only the control group exhibits the typical: (i) *global precedence effect*, *w*ith faster responses to global than to local identity judgments, and (ii) the *inter-level interference effect*, with greater effects of consistency on local compared with global identity trials. In fact, for the DP group there is hardly any difference between local and global consistent trials (8 ms), and their global-to-local interference effect is not larger than the local-to-global interference effect. Hence, there are two aspects where the DP group differs from the control group: (i) they show no global precedence effect, and (ii) they exhibit local-to-global and global-to-local interference effects of similar magnitudes. The former effect was also reflected in the Global-Local Precedence index where the DP group obtained reliably lower scores than the control group.

If the lack of a global precedence effect in the DP group reflects delayed derivation of global shape information, what then is the cause of this impairment? We see two possibilities; an attentional and a perceptual one. If attention is not evenly spread across visual space, but is somehow restricted, local shape information may be derived as quickly as global shape information. We will examine this possibility more stringently in the next experiment. If we find no evidence of restricted allocation of attention in the DP group, this will suggest that the impaired derivation of global shape information reflects a perceptual rather than an attentional deficit.

## TVA-based assessment of attentional functions

The Theory of Visual Attention (TVA) [34] is a formal computational framework offering a quantitative analysis of individual differences in attentional functions. TVA assumes that visual categorizations of objects in the visual field compete (race) for access to a visual short-term memory (VSTM) with a limited storage capacity of *K* objects. The processing rate at which an object *x* races toward VSTM is given by,

$$v_{x}\;=\;C\frac{w_{x}}{\sum_{z\in S}^{}w_{z}}$$

where *C* is the total processing capacity and $w_{x}/\sum_{z\in S}^{}w_{z}$ is the relative attentional weight of object *x* (i.e., the attentional weight of object *x* divided by the sum of attentional weights across all objects in the visual field, *S*). That is, objects with high attentional weights will be more likely to access the limited VSTM storage than objects with low attentional weights.

Depending on the specific TVA-based experimental paradigm used, different parameters quantifying attentional functions can be derived. Here, we employed the CombiTVA paradigm (a combination of a whole report and a partial report paradigm; Vangkilde, Bundesen [35]) from which five distinct components of attention can be estimated: (1) *K*, the capacity of VSTM measured in number of letters; (2) *C*, the total processing capacity measured in letters per second; (3) *t*_0_, the temporal threshold of conscious perception measured in milliseconds; (4) α, the top–down controlled selectivity defined as the ratio between the attentional weight of a distractor *w*_*D*_ and the attentional weight of a target *w*_*T*_ (i.e., *α* = *w*_*D*_/*w*_*T*_); and finally (5) *w*_index_, the laterality index of attentional weights defined as the ratio between the sum of attentional weights assigned to objects in the left hemifield and the sum of attentional weights across the entire visual field.

*K* is assumed to vary on a trial-by-trial basis. Thus, the *K* value is the expected *K* given a particular probability distribution (i.e., the probabilities that on a given trial *K* = 1, 2,…, 6). The *w*_index_ is based on six attentional weights, one for each letter position.

An α value close to zero reflects efficient selection of targets whereas a value close to 1 indicates no prioritizing of targets compared with distractors. Thus, if individuals with DP have a problem in attentional selection this will result in a larger α value for these individuals compared with control subjects. The laterality index, *w*_index_, will help to assess if individuals with DP spread attention differently across the visual field than control subjects. A laterality index of 0.5 indicates an equal weighting of the left and right visual field, whereas a value closer to 0 reflects a right-sided bias and a value closer to 1 reflects a left-sided bias. To further quantify the participants’ spatial distribution of attention, we also calculate the standard deviations of the relative attentional weights, *SD*_weight_ [36] where a value of zero indicates that attention is distributed equally on all the six positions in the experimental paradigm, whereas a value approaching $\sqrt{1/6}$ reflects a very unequal distribution of attention weights.

### Method

#### Participants

The DPs’ performance in the CombiTVA paradigm was compared with 20 control participants, two for each individual with DP, matched for age and gender. These control participants were the same as the ones who served as control participants in Gerlach, Klargaard [4].

#### Stimuli

For a given trial, stimuli were chosen randomly without replacement from a set of 20 capital letters (ABDEFGHJKLMNOPRSTVXZ) written in the font Arial (broad) with a letter point size of 68 corresponding to 2.7° × 2.3° of visual angle. Target stimuli were in red and distractors were in blue. Masks were made from red and blue letter fragments completely covering the letters.

#### Procedure

**Fig 2:**
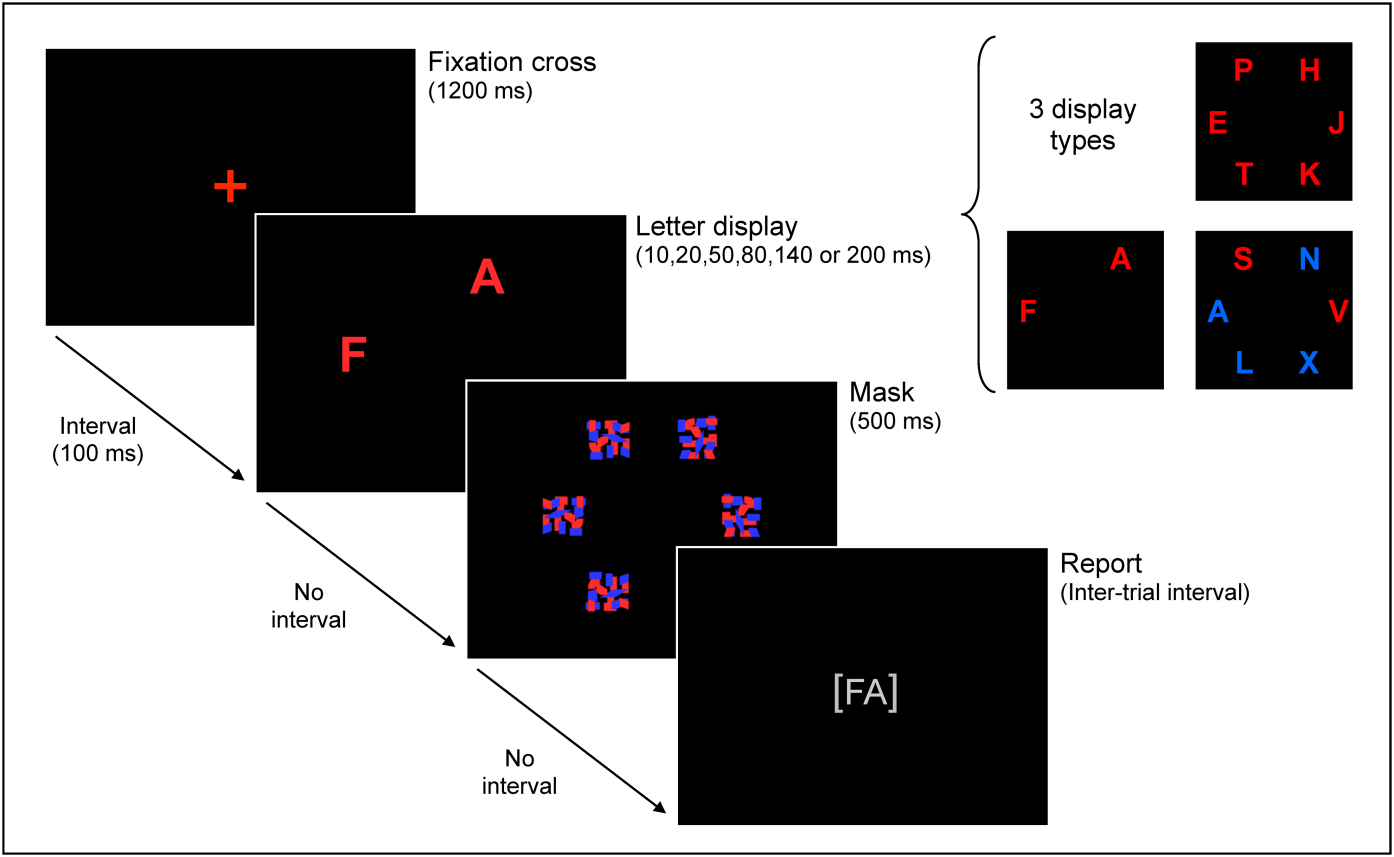
Illustration of the trial outline of the CombiTVA paradigm used to test attentional functions.

Fig 2 shows the trial outline of the CombiTVA paradigm. A trial was initiated by a red fixation cross presented in the middle of a black screen for 1200 ms. After a 100-ms blank screen, either six red target letters, two red target letters, or two red target letters and four blue distractor letters were presented briefly on an imaginary circle with a radius of 7.5° of visual angle. Letter displays containing six target letters were presented for 10, 20, 50, 80, 140, or 200 ms, whereas all other types of letter displays were presented for 80 ms. Letter displays were terminated by pattern masks presented for 500 ms on all possible stimulus positions, where after participants made an unspeeded report of all the red target letters they were ‘fairly certain’ of having seen. Participants reported by typing the letters in any order on a standard keyboard. After each block, participants were informed about the accuracy of their responses and were instructed to aim for response accuracy between 80 and 90%. The paradigm was presented on a 19” CRT monitor running at a refresh rate of 100 Hz. One individual with DP and one control performed the paradigm with a refresh rate of 85 Hz resulting in slightly longer presentation durations (e.g., 11.76 ms instead of 10 ms). These data were included in the analysis because the applied modeling procedure took the actual presentation duration into account, so that estimations of the attentional components should not be affected.

#### Design

The CombiTVA paradigm comprised 24 practice trials and nine experimental blocks of 36 trials. Within an experimental block, 18 trials contained six target letters (three trials for each of the six exposure durations), nine trials contained two target letters, and nine trials contained two target letters and four distractor letters.

### Results and discussion

For each participant, TVA-parameters were estimated by a maximum-likelihood procedure using the LibTVA toolbox [37]. Table 2 shows the mean of the estimated parameters for individuals with DP and control participants. No significant differences were found for any of the five estimated parameters or the error rate when comparing the two groups (all *p*’s > .11; see Table 2), thus top–down controlled selectivity quantified by the *α* parameter does not seem to differ between individuals with DP and control participants making it unlikely that the deviant performance of the DP group in Navon’s paradigm is related to a problem in attentional selection. Neither did we observe any difference in how individuals with DP allocate spatial attention in comparison with control participants, which was quantified by the laterality index, *w*_index_ (*p* =. 11), and the standard deviation of the relative attentional weights, *SD*_weights_ (*p* = .41). Together, these findings suggest that the impaired derivation of global shape information observed in individuals with DP reflects a perceptual rather than an attentional deficit. For additional information regarding the individual TVA parameters see S2 Dataset.

**Table 2:** TVA-parameter estimates of attentional functions.

	*K*	*C*	*t*_0_	α	*w*_index_	*SD*_weights_	Error rate
**DPs**	3.04 (0.66)	54.8 (16.6)	19.4 (13.3)	0.88 (0.35)	0.61 (0.13)	0.15 (0.06)	0.25 (0.08)
**Controls**	3.06 (0.90)	58.9 (20.6)	15.6 (8.2)	0.76 (0.38)	0.54 (0.10)	0.13 (0.05)	0.2 (0.11)
***p*-value**	.94	.60	.42	.42	.11	.41	.22

Mean TVA-parameter estimates and SD (in brackets) for the group of individuals with developmental prosopagnosia (DP) and the control group (Controls). Also given are the *p*-values associated with the difference between DPs and Controls for the TVA-parameter estimates (based on independent samples *t*-tests).Units for the individual parameters are *t*_*0*_ (ms), *C* (letters/second), *K* (letters), *α* ranges from perfect selection at 0 to non-selectivity at 1, *w*_*index*_ ranges from complete rightward bias at 0 to complete leftward bias at 1 with 0.5 indicating equal weighting between the two visual fields, *SD*_*weights*_ ranges from equal weighting on all six locations at 0 to all weight on a single location at $\sqrt{1/6}$.

## The relationship between local/global processing and recognition of faces and objects

Having established that the DP group do exhibit an unusual performance (no global precedence effect) in Navon’s paradigm compared with the control group, we next examined whether this deficit might be systematically related to the DPs’ face and object recognition performance.

### Face recognition

#### Method

To examine whether face recognition performance varied systematically with the DPs’ performance in the Navon paradigm, we performed a correlation analysis based on the Global-Local Precedence index derived from the Navon paradigm (the standardized mean difference (Cohen’s *d*) between RTs to Local and Global Consistent trials) and face recognition performance in the CFMT (number of correct responses). This was done by means of Pearson’s correlation coefficient (*r*). We report one-tailed statistics because we test a directional hypothesis: The lower the values on the Global-Local Precedence index, the poorer the performance on the CFMT.

#### Results

**Fig 3:**
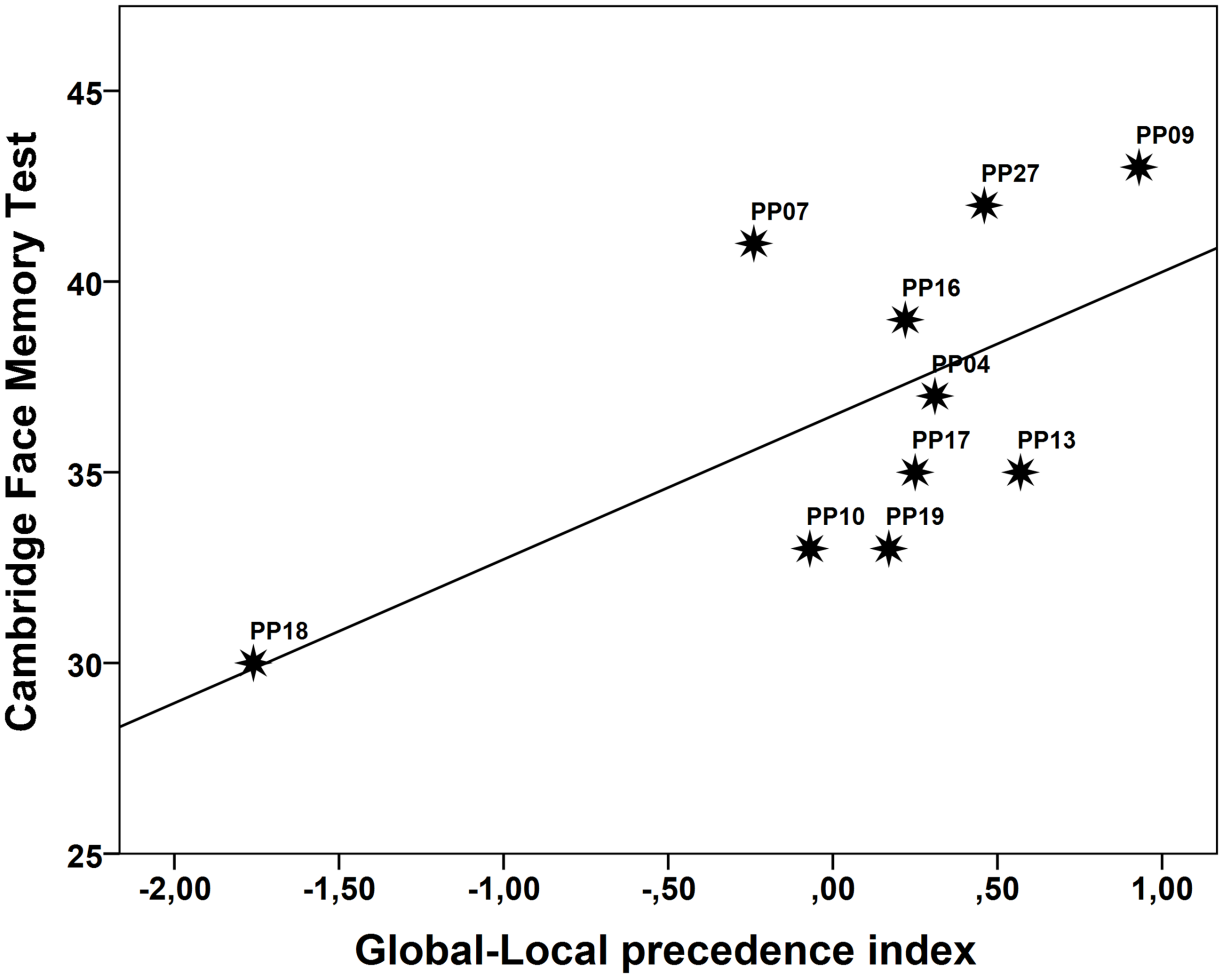
Scatterplot showing the relationship between performance on the Cambridge Face Memory Test (number of correct responses) and the Global-Local Precedence index in the DP group.

The analysis revealed a significant correlation between the Global-Local Precedence index and performance on the CFMT (*r* = .63, *p* = .026); the lower the score on the Global-Local Precedence index the poorer the performance on the CFMT (see Fig 3 and also Dataset S2 Dataset for information regarding the individual scores on the CFMT). To examine the credibility of the correlation we estimated the one-tailed lower bound of the 95% CI of the correlation by means of bias corrected bootstrap analysis (1000 samples). This yielded a value of -.06, suggesting that the sample correlation is not a reliable estimate of the relationship in the population of DPs at large. Because of this we ran additional regression diagnostics on the relationship between the Global-Local Precedence index and CFMT performance. This revealed that none of the standardized residuals had a value larger than 1.52 suggesting that the regression model fitted the data well. Also, for no case was Cook’s distance larger than .18 which also suggests that no single case had a critically high influence on the model as a whole. However, PP18’s score on the Global-Local Precedence index did deviate significantly from the others (Mahalanobis distance = 6.5, *p* < .01). In sum, PP18 clearly departs from the other DPs in her Global-Local Precedence effect (*Z* = -2.68), but it is not the case that she influences the model unduly because she also scores comparably lower on the CFMT (*Z* = -1.65). As such, she seems to occupy the extreme end of a continuum. This raises the question of whether she should be excluded from the analysis altogether. Considering that we have no reason to doubt the validity of her data—both the CFMT and the Global-Local Precedence Index have been shown to be reliable measures, and PP18 performed the Navon task quite accurately (97% correct)–we also find no reason to exclude her. Nevertheless, if she is excluded the correlation is reduced and no longer significant (*r* = .37, *p* = .17); an aspect also reflected by the lower bound of the 95% CI being -.06. In conclusion, while we have no reason to doubt the correlation observed in the present sample, it does not provide a reliable estimate of the relationship between global/local processing and face recognition in the population of DPs at large. Accordingly, the generalisability of this particular finding must await confirmation by other studies.

#### Discussion

We find a systematic relationship between the Global-Local Precedence index and face recognition performance; the lower the global precedence effect the poorer the face recognition performance. This suggests that differences in the global precedence effect may be sufficient to drive the relationship between performance in the Navon paradigm and face recognition performance; it does not necessarily reflect interference effects.While this pattern was significant for the present sample of DPs, we note that the sample correlation is not a reliable estimate of the relationship in the population of DPs at large.

### Object recognition

Given the relationship found between face recognition ability and differences in the Global-Local Precedence index in the present sample of DPs, we next examined if such a relationship might extend to their visual object recognition performance. Our motivation for examining this aspect is that we have previously found a high correlation between performance on the CFMT and performance on visual object recognition tasks for all the DPs examined here [4]. Of special interest in the present context was the finding that the group of DPs was more affected by stimulus degradation (presenting objects as silhouettes and fragmented forms) than the control group (for data from the individual DPs and controls in the object decision tasks we refer to the supplementary material provided in [4]). This relates to the present observations based on the Navon paradigm because recognition of silhouettes and fragmented forms is assumed to be particularly dependent on global shape processing [38, 39]. Based on this we thus predicted a positive correlation between the Global-Local Precedence index and recognition performance with silhouettes and fragmented forms in our group of DPs.

#### Method

Gerlach, Klargaard [4] assessed visual object recognition performance by means of object decision tasks, where participants had to decide whether the stimuli depicted real objects or nonobjects. In these tasks, the nonobjects are chimeric, consisting of parts of two real objects, which makes the task quite demanding in terms of perceptual differentiation. There were three versions: One with regular line drawings, one with silhouette drawings, and one with fragmented drawings (for further details see Gerlach, Klargaard [4]). Performance was measured in terms of *A* [40] which is a bias-free measure that varies between 0.5 and 1.0 with higher scores indicating higher sensitivity, that is, better discrimination between objects and nonobjects. We performed correlation analyses based on the scores on the Global-Local Precedence index and performance with silhouettes and fragmented drawings respectively. We report one-tailed statistics as we test a directional hypothesis; the lower the values on the Global-Local Precedence index, the poorer the discrimination sensitivity on the object decision tasks with silhouettes and fragmented forms. Two of the DPs did not contribute to the analysis based on object decision with silhouettes (PP16 did not perform the task and PP04’s data could not be interpreted due to chance level performance with extreme hit and false alarm rates, see Gerlach, Klargaard [4]).

#### Results

The comparison of scores on the Global-Local Precedence index and performance with silhouette drawings revealed a significant correlation (*r* = .72, *p* = .022; one-tailed lower bound of the 95% CI = .13 estimated by means of bias corrected bootstrap analysis (1000 samples)), as did the comparison between scores on the Global-Local Precedence index and performance with fragmented drawings (*r* = .72, *p* = .01; one-tailed lower bound of the 95% CI = .28 estimated by means of bias corrected bootstrap analysis (1000 samples)). In both cases, lower values on the Global-Local Precedence index were associated with poorer recognition of degraded stimuli.

**Fig 4:**
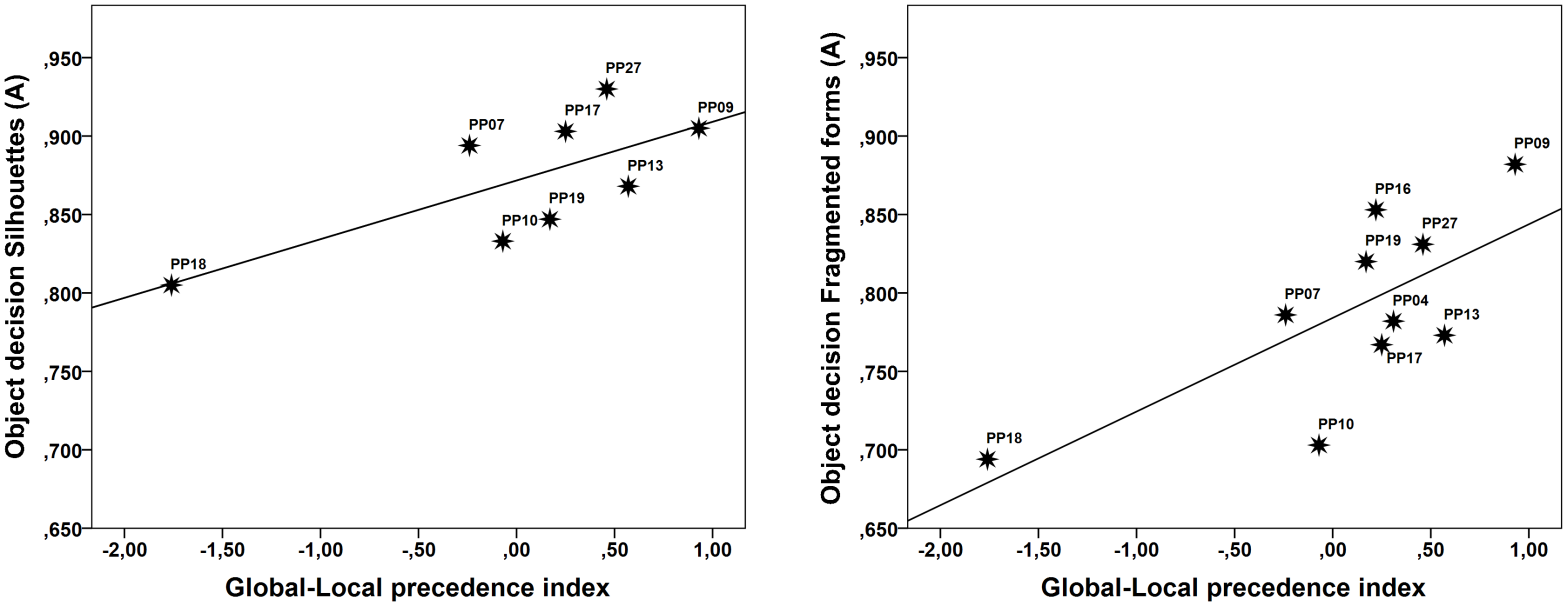
Scatterplots showing the relationship between scores on the Global-Local Precedence index and performance with recognition of silhouettes and fragmented forms in the DP group.

From Fig 4, showing scatterplots of the relationship between scores on the Global-Local Precedence index and performances in the object decision tasks with silhouettes and fragmented forms, it can be seen that PP18 again sticks out, just as in the former analysis involving face recognition performance. This is not surprising considering that the Global-Local Precedence index is the same. While PP18 clearly do add to the variability in the data, the lower bounds of the 95% CIs associated with the correlation did not include 0. Hence, while the observed correlations between the Global-Local Precedence index and performance with silhouettes and fragmented forms are not very precise estimates of what can be expected in the general population of DPs, the findings do seem generalizable. See also S3 Dataset for additional information regarding the individual scores on object decision with silhouettes and fragmented forms.

#### Discussion

The positive correlations between the Global-Local Precedence index and object recognition performance with silhouettes and fragmented drawings suggest that delayed global shape processing (a reduced global precedence effect) impacts negatively on the recognition of degraded material. Accordingly, the prediction regarding an important role of global shape processing in recognition of degraded material [38, 39] was confirmed.

## General discussion

There seems to be a growing consensus that a central deficit underlying the face recognition impairment in developmental prosopagnosia (DP) reflects deficient holistic processing. Evidence supporting this notion comes from several lines of inquiry, and in particular from studies showing that individuals with DP perform abnormally in paradigms taken to reflect holistic processing in normal participants: The Garner speeded-classification task with faces [11], and the Composite Face Task [2, 13]. Common for both paradigms is that individuals with DP often do not exhibit the same interference effects as normal participants. This suggests that individuals with DP do not simultaneously or holistically process the various types of information contained in faces (features and the configuration of these features). A related paradigm, which has also been used in the study of DP, is that of Navon [23]. Even though this paradigm is typically used with compound letters rather than faces, it also measures configural processing (the formation of a global shape based on local elements). It is thus a reasonable hypothesis that individuals with DP may also exhibit impaired performance in the Navon paradigm; at least if their face recognition problem reflects impaired configural processing [22]. Consistent with this, a correlation between face processing performance and performance on the Navon paradigm in individuals with DP has been reported [2, 22, 28]. However, because these studies have used different indexes of global/local processing, it is not clear which mechanism(s) has(have) been driving the observed correlations; is it global/local precedence effects, which tell us something about which type of information is processed first (global or local), or is it interference effects, which reflect what level of information is weighted the most when shape information from different spatial scales are compared?

To answer this question we examined the relationship between face recognition performance, as measured with the Cambridge Face Memory Test (CFMT), and a simple index of Global-Local precedence bias based on the standardized mean difference (Cohen’s *d*) between RTs to Local and Global Consistent trials in 10 individuals with developmental prosopagnosia (DP). This analysis revealed a systematic relationship (*r* = .63); the lower the global precedence effect, the poorer the face recognition performance.

It was not the case, however, that the DPs could not derive the global shape at all. Indeed, they did not make more errors than the control participants, and they also exhibited a global-to-local interference effect. This would not be expected if the individuals with DP were unable to identify the configuration of the local elements; i.e. the global shape, at all. Hence, the problem for the DPs seems to be a *delay* in the derivation of global shape information. For the DPs the average difference between Local and Global Consistent trials was 8 ms whereas it was 48 ms for the control participants. It is important to note, that this difference between DPs and control participants does not reflect a general attentional deficit, as the attentional capacities of our sample of DPs were found to be normal. Accordingly, the problem exhibited by the DPs is likely to reflect a perceptual rather than an attentional deficit.

We have also examined the visual object recognition ability in our group of DPs using demanding object decision tests (deciding whether pictures represent real objects or nonobjects) with regular line-drawings, silhouettes, and fragmented drawings [4]. The DP group was impaired on these tasks when the stimuli were degraded (silhouettes or fragmented drawings). In addition, their face recognition performance, as assessed with the CFMT, correlated significantly with their object recognition performance with both silhouettes (*r* = .87) and fragmented forms (*r* = .78) [4].

The relationship between performance in Navon’s paradigm and performance on the CFMT on the one hand, and the close relationship between performance on the CFMT and performance with degraded material in the visual object recognition tasks on the other, prompted us to examine here whether the DPs’ visual object recognition performance with degraded material would also correlate with the Global-Local Precedence index of the Navon paradigm. We deemed such a relationship likely because recognition of silhouettes and fragmented forms is assumed to place particular demands on global shape processing [38, 39]. This prediction was borne out as we find reliable relationships between scores on the Global-Local Precedence index and performance with silhouettes and fragmented forms. The lower the global precedence effect (low values on the Global-Local Precedence index), the poorer the performance with silhouettes and fragmented forms.

The systematic relationships between impaired performance in Navon’s paradigm, the CFMT, and object recognition tasks with degraded material in the same group of individuals with DP, suggest that these deficits could very well reflect dysfunction of the same underlying mechanism. As shown above, these impairments are observed in DPs who have no general visual attentional deficits, which can explain their impaired performance. This leads us to conclude that the common underlying deficit is perceptual in nature, and that it reflects delayed derivation of global relative to local shape information.

Even though the present findings suggest a common denominator, the pieces of evidence presented in favour of it are not equally strong. While the relationship between the Global-Local Precedence index and object recognition performance with both silhouettes and fragmented forms were robust, as was the reduction in the global precedence effect in the DP group, the relationship between the Global-Local Precedence index and face recognition performance was not. Hence, while the regression model provided a good fit of the data, and with none of the cases having undue influence on it, the correlation observed was not a reliable estimate of such a relationship in the population of DPs at large. This was due to one of the DPs (PP18) scoring extremely low on the Global-Local Precedence index (but also rather low on the outcome variable). Whether the performance of this particular case represents a valid observation from the extreme end of a continuum, or whether it represents an anomaly, can only be settled by future studies. We note, however, that both the CFMT and the Global-Local Precedence Index have been shown to be reliable measures, and PP18 performed the Navon task quite accurately (97% correct), which suggests that PP18 represents a valid case. Despite the uncertainty regarding this particular aspect of our results, we argue that when the pieces of evidence presented here are considered as a whole, they fit together both theoretically and with other lines of evidence.

The suggestion that DPs may exhibit delayed processing of global shape information is consistent with recent findings by Awasthi, Friedman [41]. They have shown that interference effects—owing to incongruent information originating from low and high spatial frequency components—occur much later in time in individuals with DP compared with control participants in a face gender identification task. While these authors could not conclude for certain whether this performance pattern was due to an integration deficit or to slower processing of low spatial frequency components, our findings support the latter interpretation; granted of course that global shape is mediated by low spatial frequency components. What our results show in addition is that this deficit: (i) is perceptual rather than attentional, (ii) seems to be directly related to face recognition performance (individuation), and (iii) also affects recognition of other non-face stimuli for which global shape is critical. The present interpretation also fits with a recent demonstration that the EEG-based event-related N250 component, taken to be a marker of the matching between a seen face and visual long-term memory (VLTM) representations, was delayed by approximately 40 ms in a group of DPs compared with controls [42]. In comparison, the earlier N170 component, which is enhanced to faces compared with non-face objects, and believed to reflect early perceptual encoding, have not been shown to be delayed in DP [43]. Even so, the N170 is atypical in DP (being just as large to inverted as to upright faces), and it has been implied that the delayed N250 in DPs may in fact reflect early face processing abnormalities associated with the atypical N170 component; a problem with encoding facial features relative to the spatial layout of canonical (upright) face templates [43].

An explanation of why delayed global shape processing may cause impaired recognition—and in particular impaired recognition of faces—can be found in the PACE (Pre-semantic Account of Category-effects) model proposed by Gerlach [38]. This model assumes the existence of two operations in visual object recognition: *shape configuration* and *selection*. Shape configuration refers to the binding of visual elements into elaborate shape descriptions corresponding to whole objects or large object parts; descriptions in which relationships between the parts are specified. The configured shape representation is a description that can be matched with structural representations of whole objects or large object parts stored in VLTM. The matching process is thought of as a race among VLTM representations that compete for selection. The VLTM representation that matches the configured representation the best will win the competition; hence be selected. The match criterion is task dependent. If the task requires fine-grained discrimination, a strict match criterion will be in place. If the task only requires gross perceptual processing, a more lax match criterion will be sufficient. When the configured representation is successfully matched with VLTM representations according to a given criterion, the object is classified as a particular sort of instance. In PACE, shape configuration does not precede access to stored visual knowledge. Rather, shape configuration follows a first pass access to VLTM representations based on processing of global shape information (outline shape) in the visual input. This first pass yields initial hypotheses concerning the likely identity of the stimulus. These hypotheses are then used in a top-down manner to augment the buildup of a more detailed description of the stimulus (i.e. shape configuration), which again serves as input for a more specific match with VLTM representations [38, 39, 44]. The greater the demand placed on perceptual differentiation, the more loops comprising VLTM access → shape configuration may be required to reach a successful match between the visual input and VLTM representations (i.e. recognition). From this description it is clear that fast derivation of global relative to local shape information is rather important in the recognition process because it facilitates the matching process by narrowing down the scope of likely VLTM candidates, but also because it provides the initial frame in which local details can later be embedded [45, 46]. Hence, when interpreted in the PACE framework it makes good sense that delayed derivation of global shape will not only slow down performance, but also lead to recognition problems. In particular, we have previously made the case that derivation of global shape is especially important for recognizing objects characterized by a high degree of visual similarity when the demand for perceptual differentiation is high [38, 39]. Face recognition seem to be characterized by both of these aspects: Faces are highly visually similar and they are typically recognized at a subordinate level which requires more perceptual differentiation than for example basic level recognition. With regards to visual similarity we note that the DPs examined here have previously been shown to be more affected by increasing levels of visual similarity than control subjects when processing faces [4]. Finally, given that degraded objects are also likely to place high demands on global shape processing [38, 39] it may not be a chance finding that recognition of faces and degraded objects are found to be systematically related with each other and with a reduced global precedence effect in our DP sample.

As argued above, the individuals with DP that we have studied do not necessarily have a problem with the integration of features per se; indeed they are able to derive the overall configuration of elements that are spatially separated (they can identify letters at the global level in the Navon paradigm; it just happens more slowly than in controls). At first glance this may seem at odds with the observation that DPs often do not exhibit interferences effect in the Composite Face Task and in Garner’s speeded-classification paradigm. This, however, could be a consequence of their reduced global precedence effect. If global shape representations are not accessed early in the recognition process it is likely that this will also affect the matching of the visual input to VLTM representations so that matches based on features or individual second-order relations (e.g., the distance between the eyes) are weighted more heavily than is the overall gestalt. Consequently, it may be easier for individuals with DP to selectively process face parts—a sort of release from global binding—making them less susceptible to interference effects in the Garner and the Composite Face paradigms. The idea that a reduced global precedence effect may also cause local shape information to be weighted just as much as—or even more than—global shape information in the matching process is in accord with the Navon performance of the DPs we have examined here. They showed global-to-local and local-to-global interference effects of similar magnitude whereas the control participants showed a significantly larger global-to-local than local-to-global interference effect.

The PACE hypothesis concerning the importance of global shape based top-down processing effects in face recognition bears some resemblance to the Perceptual field hypothesis suggested by Rossion [47] to explain the Composite Face effect and other phenomena in face recognition. According to Rossion “…a holistic (upright) face representation acts as a template and is necessary to guide the perception of the simple elements of an incoming visual face stimulus, in a top–down manner. Hence, the features of an upright and an inverted face would not be perceived the same way, because the inverted face could not benefit from this template matching.” (Rossion [48], p. 308). Also, like in the PACE model, what characterizes face recognition according to the Perceptual field hypothesis is that faces are processed “…*at* a sufficiently fine-grained level of resolution to individualize members of the face class” (Rossion [21], p. 153). What seems to differ between the hypotheses is that PACE makes no assumption regarding a qualitative difference in the type of processing that faces and objects undergo. The difference is quantitative and depends on the visual similarity of the objects and the level of recognition required (superordinate → subordinate).

Even though the assumption of delayed derivation of global shape in DP seems to offer good explanatory power in accounting for face recognition problems in DP, we do not suggest that this impairment is the sole explanation for the severely disrupted face recognition abilities of people with DP. After all, the Global-Local Precedence index could ‘only’ account for 40% of the variance in the face recognition performance in the DPs studied here. The same is true of the relationship between Navon performance and visual object recognition performance with degraded stimuli. Here, the Global-Local Precedence index could explain approximately 52% of the variance in recognition of silhouettes and fragmented forms. Accordingly, even in the DP group studied here, not all of the variance in face and object recognition performance can be accounted for by differences in global precedence effects. Nevertheless, we do believe to have demonstrated that delayed global shape processing may contribute significantly to face recognition problems, and that the PACE model offers a fruitful framework for understanding why this may be the case.

## Supporting information

S1 Dataset Individual data for developmental prosopagnosics and control participants in Navon’s paradigm. (SAV)

S2 Dataset TVA parameter estimates for developmental prosopagnosics and control participants. (SAV)

S3 Dataset Individual data for developmental prosopagnosics for the Cambridge Face Memory Test (CFMT), the Global-Local Precedence Index, performance on object decision with silhouettes, and performance on object decision with fragmented forms. (SAV)
